# Presence of antimicrobial resistance genes in biofilms from swine drinking water pipes before and after treatment with peracetic acid

**DOI:** 10.3389/fmicb.2026.1770950

**Published:** 2026-04-29

**Authors:** Gabrielle E. Doughan, Becca K. Walthart, Cora E. Schau, Kristin J. Skoland, Kathy T. Y. Mou, Justin T. Brown, Jessica L. Bonnema, Paul J. Plummer, Danyang Zhang, Ganwu Li, Locke A. Karriker

**Affiliations:** 1 Department of Veterinary Diagnostic and Production Animal Medicine, Swine Medicine Education Center, College of Veterinary Medicine, Iowa State University, Ames, IA, United States; 2 Department of Veterinary Diagnostic and Production Animal Medicine, Veterinary Diagnostic Laboratory, College of Veterinary Medicine, Iowa State University, Ames, IA, United States; 3 College of Veterinary Medicine, University of Tennessee, Knoxville, TN, United States; 4 Department of Statistics, Iowa State University, Ames, IA, United States

**Keywords:** AMR, ARGS, biofilm, integron genes, peracetic acid, swine, water lines

## Abstract

Biofilms can be problematic to swine drinking water systems as they can harbor pathogens, decrease water quality, and may contribute to antimicrobial treatment failure. Water-administered antimicrobials are used for disease treatment in swine populations, yet, little is known about water line ecology and the impact it can have on antimicrobial resistance and stewardship. Water line cleaning and disinfection may aid in removal of water line biofilms, improve swine health, and antimicrobial stewardship. Water line samples were collected pre-treatment (0), 24 h post-treatment with 0.78% CID 2000 Pro (peracetic acid) (1), and 3, 5, 7, 14, 21, 42, 56, and 77-days post-treatment from six wean-to-finish swine farms in Iowa, USA. Biofilm was aseptically extracted from the interior of the water line pipe (*n* = 119) and submitted for metagenomic analysis to detect antimicrobial resistance genes (ARGs). This study demonstrates high prevalence of ARGs in swine water line biofilms that could confer resistance to both medically important antimicrobials to humans and animals such as aminoglycosides, beta-lactams, fluoroquinolones, colistin, and fosfomycin. From 115 samples, a frequency of 3,904 ARGs were reported, with 184 unique ARGs defined. Four samples contained no ARGs. One hundred and fifty-one integron genes representing three classes were found in 115 of 119 samples, indicating mechanisms of potential spread of multiple drug resistance. ARGs and integron genes combined were significantly lower on average by 10 unique ARGs/ integron genes 24-h post-treatment (1) when compared to pre-treatment (0) counts (*p*-value = 0.01). The number of unique ARG and integron genes quickly rebounded and were not statistically significant compared to pre-treatment counts on post-treatment dates 3, 5, and 7 (adjusted *p*-value ≥ 0.05), and by post-treatment date 14, unique ARG and integron genes were significantly higher than pre-treatment (adjusted *p*-value = 0.012). This study demonstrates that swine water line biofilms can harbor antimicrobial resistance genes which could have potential clinical impacts on pig health and treatment response.

## 1 Introduction

Antimicrobial resistance (AMR) is a vital One Health concern affecting human, animal, and environmental health. Monitoring antimicrobial use and resistance is critical to identify risks and strategies for antimicrobial management and stewardship (Góchez et al., 2019; Spronk et al., 2023). In livestock production, antimicrobial use remains essential for treating, controlling, and preventing disease, but can also be associated with the development and dissemination of antimicrobial resistance genes (ARGs) and antibiotic-resistant bacteria (ARB) (He et al., 2019; Lei et al., 2024; Xu et al., 2022). Administration of antimicrobials via the drinking water is a critical tool for treatment, prevention, and control of disease in population veterinary medicine (Lekagul et al., 2019; U.S. Department of Agriculture, 2019). A survey by the U.S. Department of Agriculture (2019) reported 60–80 percent of surveyed growing pig sites (nursery, wean-to-finish, and grower sites) from 13 top pork producing states administered one or more water antimicrobials during the growing stage, including the use of medically important antimicrobials. Antimicrobials used in the reported survey included tetracyclines, beta-lactams, aminoglycosides, and lincosamides (U.S. Department of Agriculture, 2019). Efforts in improving swine water distribution system design (Little et al., 2021a) and water medication management to improve treatment and drug efficacy have been made (Decundo et al., 2019; Edwards, 2018; European Medicines Agency, 2022; Georgaki et al., 2023; Hawkins and Temko, 2010; Kotb et al., 2019; Little et al., 2021b; Münster and Kemper, 2024; Smith et al., 2024), however, the role of biofilms and their contribution to AMR in swine drinking water systems is understudied (Doughan et al., 2022; Irshad et al., 2025). A recent report found ARGs in swine water line biofilm samples associated with resistance to multiple, medically important drug classes and this warrants further study of the frequency in other farms (Doughan et al., 2022; Scott et al., 2019).

Evaluating genotypic resistance through the presence of ARGs often utilizes technologies such as metagenomics techniques or quantitative polymerase chain reactions (qPCR) (de Abreu et al., 2021; Li et al., 2021). Other technologies are available for characterizing ARGs, host relationships with presence of ARGs, and phenotypic resistance evaluation which have been described elsewhere (Kaprou et al., 2021; Rice et al., 2020). Biofilms are well known to facilitate dissemination of resistance through horizontal gene transfer (HGT) (de Abreu et al., 2021). Biofilms are often heterogenous communities of bacteria embedded in extracellular polymeric substance (EPS) in which the organisms are in close proximity to one another. This provides opportunities for transfer of ARGs through conjugation (de Abreu et al., 2021; Olsen, 2015). Transduction, transformation, and specific point mutations are other potential mechanisms of ARG spread (Corona and Martinez, 2013; de Abreu et al., 2021; Olsen, 2015). Comprehensive reviews describing the mechanisms for development of antimicrobial resistance are available (Lahiri et al., 2021; Munita and Arias, 2016; Olsen, 2015; Uruén et al., 2020). Antimicrobial resistance genes that are spread via HGT involve the transfer of plasmids and transposons carrying ARGs between bacterial organisms, which can also transfer multiple ARGs to contribute to the spread of multi-drug resistance (Deng et al., 2015; Mazel, 2006). Integrons are genetic elements found in plasmids and transposons which facilitate the dissemination of ARGs by incorporating gene cassettes into other integrons or within bacterial genomes (Deng et al., 2015). Therefore, the study of the presence of integrons can also provide useful insights into the mechanisms of spread of multiple drug resistance. Characterizing inheritable mechanisms of resistance in swine water line biofilms is vital for identifying areas for mitigation strategies and improved antimicrobial stewardship efforts.

Removing biofilm from water lines may reduce the incidence of ARGs and could result in improving water quality and the further spread of AMR (Davies and Wales, 2019). Peracetic acid (PAA) is a strong oxidant with broad-spectrum disinfection capabilities against various microorganisms and works efficiently at removing biofilms (Farjami et al., 2022; Kampf, 2024; Kitis, 2004; Zhang et al., 2022). It has been used for water and wastewater treatment due to its minimal risk of toxic by-products (Kitis, 2004). Alternatively, other evidence suggests that exposing bacteria to biocides or their improper application [e.g., triclosan (Curiao et al., 2016), chlorhexidine (Curiao et al., 2016), aldehydes, quaternary ammonium compounds, etc., (Webber et al., 2015)] could select for co-resistance to antimicrobials (Davies and Wales, 2019; Maillard, 2018). CID 2000 Pro (CID LINES, an Ecolab Company, Ghent, Belgium) is a commercially available PAA and can be utilized between groups of pigs for water line cleaning and disinfection. Label indications state to apply a 2% dose to clean and disinfect water lines, however most swine farms are equipped with a fixed dosing medicator of 0.78%. The impacts on ARGs are unknown if PAA is applied in water lines as at this lower dose.

The objectives of this study were to confirm the presence and characterize the types of ARGs and integron genes in swine farm water systems and to evaluate the presence of ARGs before and after a one-time water line cleaning with 0.78% peracetic acid. The null hypothesis is that ARGs and integron genes are not statistically different before and after a one-time administration of 0.78% PAA in swine water lines.

## 2 Materials and methods

### 2.1 Study design

An observational, longitudinal study assessed the presence of ARGs and integron genes before and after water line administration of CID 2000 Pro (CID LINES, an Ecolab Company, Ghent, Belgium), a commercially available peracetic acid (PAA) formulation, in six commercial wean-to-finish pig operations. Each of the farms were sourced on individual, private well water. Each farm contained two rooms which were identified by navigational direction. This research was performed as a part of a larger study completed by Doughan (2025). One 7.62 cm × 1.905 cm internal diameter polyvinyl chloride (PVC) water pipe sample was collected at each time point from each room within a site. Water line samples were collected across 10 time points over a 78-days period from two rooms in each site. Initial samples were collected prior to administering CID 2000 Pro (PAA) at 1:128 oz. The water lines were filled with PAA and allowed a 24-h contact time while the facilities were free of pigs. Samples were collected at pre-treatment (0), after 24 h of contact time followed by a fresh water flush (1), then 3, 5, 7, 14, 21, 42, 56, 77 days post-treatment. The barns were repopulated with pigs after water line treatment and freshwater flush. Water line samples were collected during May – November 2023.

### 2.2 Water line coupon installation and extraction

Water line pipe coupon creation, coupon extraction, peracetic acid application and removal, sample transportation, and laboratory submission have been described in Doughan (2025) in detail. Essential methodology for this specific analysis are noted as a part of this study.

New, 1.905 cm internal diameter PVC pipe with removable PVC sections via union joinings (“coupon side streams”) were installed off of existing 1.905 cm PVC main water line pipes in six commercial wean-to-finish farms. Each coupon side stream contained five 26.67 cm removable PVC pipe sections that could be subdivided (coupons) and were designated for further testing. Two coupon side streams were installed near the middle of two rooms in each site (*n* = 12). Coupon side streams were separated into two sections to avoid cutting off water access to areas of the farm due to existing water system design. The coupon side streams contained new “T” joints, 90° elbows, and PVC ball valves that redirected farm water flow through the coupons and continued to supply the remaining water lines in the farm. Details and schematics of coupon side streams can be found in Doughan (2025). Coupon side streams were installed and allowed an acclimation period of an average of 215 days prior to the initiation of the study. At each collection time point, one, 7.62 cm PVC pipe section was collected for next generation sequencing and shotgun metagenomic analysis from one coupon side stream from each room (*n* = 120). Samples were collected from the most distal (farthest from the water source) coupon [e.g., pre-treatment (0)] to the most proximal (closest to the water source) coupon (e.g., time point 77). Details on 0.78% PAA application and verification of dose are found in Doughan (2025).

Pipe sections were collected in an aseptic manner being washed with dish soap and water to remove dust and debris until it was visually clean. The exterior pipe was marked for subdivision of the coupon pipe sections for designated testing. Gauze sheets were soaked in a 2% diluted sodium hypochlorite (bleach) solution and were used to clean the entire coupon being collected and 15.24 cm on either side of the pipe and the ceiling. The 2% bleach was also applied to the pipe cutting blade, handle, and the resting place for the pipe cutters. The bleach was allowed a 2-min contact time on the coupon and pipe cutters. New gloves were donned during the contact time. After the contact time was met, any remaining bleach was removed with a clean paper towel.

During sample collection, one sample from site three on time point 1 became contaminated and was not analyzed, leaving the total number of coupons assessed for next generation sequencing and shotgun metagenomic analysis at 119 samples.

### 2.3 Detection of ARGs and integron genes from water line biofilm organisms

One hundred and nineteen 7.62 cm sections of water line pipe coupons submerged in 30 mL of 0.9% saline in individual Whirl-Pak^®^ bags were submitted to the Iowa State University Veterinary Diagnostic Lab (ISU VDL) for next generation sequencing for shotgun metagenomic analysis to detect total presence of ARGs and integrons.

### 2.4 Processing water coupon samples for sequencing

Water line samples were frozen at −80 °C until processing. Samples were then thawed for 45 min in a biosafety cabinet. The water line sample was placed into a 250 mL Pyrex bottle with forceps while attempting to minimize disturbance of biofilm with the forceps, and the saline was poured into the same Pyrex bottle. The Pyrex bottle was held at an angle to visualize the inside of the water line coupon. Then a sterile inoculating loop was utilized to scrape biofilm off the inside of the water line coupon until most or all the biofilm was scraped off of the closest exposed end of the water line coupon. The Pyrex bottle was then capped and the bottle was vigorously vortexed for 20 s on one side. The scraping and vortexing steps were repeated until the biofilm was visually removed from one end of the coupon. The water line coupon was extracted from the Pyrex bottle with forceps and flipped over to repeat this process to the other end of the water line coupon. Once the biofilm was removed from the water line coupon, the water line coupon was removed and discarded. The supernatant from the Pyrex bottle was then transferred to a 50 mL conical tube. The 50 mL conical tube was then vortexed for 30 s, vortexing was paused for 3 s, and then the sample was vortexed for another 30 s. The conical tube was set aside for 30 min on a tube rack to allow the supernatant and sediment to separate. After 30 min, 200 μL of supernatant was collected into two wells of a clean, 96-well plate for sequencing. This process was completed for all samples. Plates were stored at −80 °C until ready for nucleic acid extraction.

#### 2.4.1 Nucleic acid extraction and measuring DNA concentration

Nucleic acid extraction was performed on 200 μL of each sample from the 96-well plate using a MagMAX Pathogen RNA/DNA Kit and a KingFisher Flex Instrument (Thermo Fisher Scientific) following manufacturer’s instructions and then eluted into 96-well plates. DNA concentration of all samples was measured using the Qubit dsDNA High Sensitivity Assay Kit and a Qubit Flex fluorometer (Thermo Fisher Scientific) following manufacturer’s instructions.

#### 2.4.2 Library preparation and sequencing

Samples were then submitted to Iowa State University’s DNA facility for library preparation using the Nextera XT DNA library preparation kit (Illumina) with dual indexing. The pooled libraries were sequenced on an Illumina NovaSeq 6000 platform, with a 500-cycle SP Reagent Kit (Illumina) to generate 250 base-pair paired-end reads.

#### 2.4.3 Antimicrobial resistance gene detection

National Center for Biotechnology Information BLASTn and SRST2 were utilized to identify ARGs and integron genes. For BLASTn, sequences were aligned to the reference database with the following criteria: a minimum 70% nucleotide identity threshold and a coverage threshold of 50%. SRST2 was utilized using default parameters to complement the analysis. Genes were identified using the ARGannot_r3 database with *mcr* genes update. ARGs and integrons were recorded as a presence or absence of a gene, not relative abundance of the genes within a sample.

### 2.5 Statistical analysis of ARGs and integron genes

Pivot tables were generated via Microsoft Excel version 2411 (Microsoft, Redmond, WA) for descriptive analysis of ARGs and integron genes combined. Total number of ARGs (including integron classes 1–3) were aggregated from raw data for each combination of site number, rooms, and collection time point with samples that had no ARGs and integron genes combined reported at 0. To detect a statistical difference between pre-and post-treatment, R v.4.3.3 was used to generate a mixed-effects linear model using the package lme4 v1.1-35.1. Site numbers were used as the random effect, and collection time points (0 and 1) were used as categorical fixed effects. Site is the experimental unit. Fisher’s exact test was used to assess the association between collection time point and ARG drug class (including integron classes) by testing the null of independence of rows (collection time point) and columns (drug class) in a contingency table. Monte Carlo simulation with 10,000 repetitions approximated the *p*-value of the Fisher’s exact test due to the large table size. For comparison of all collection time points, the total number of ARGs and integron genes combined was modeled using a mixed-effects linear model using the package lme4 v1.1-35.1. Site number was used as the random effect and collection time points (0, 1, 3, 5, 7, 14, 21, 42, 56, and 77) were used as categorical fixed effects. An analysis of variance (ANOVA) was performed to determine if there was a statistical difference among the collection time points. *Post hoc* pairwise comparisons were completed to determine the statistical differences between collection time points. *P*-values were adjusted using Tukey’s method for comparing a family of 10 estimates to control a family-wise error rate of 0.05. A *p*-value of <0.05 was utilized to determine significance.

## 3 Results

### 3.1 Presence of ARGs and integron genes

One hundred and nineteen samples were evaluated for presence of ARGs and integron genes. One hundred and fifteen out of 119 water line biofilm samples (96.64%) detected ARGs with a total abundance of 3,904 ARGs and 151 integron genes. There were 184 unique ARGs and 3 classes of integron genes, *intl1*, *intl2*, and *intl3* among the 3,904 detections. ARGs reported have been associated with 14 drug classes [aminoglycosides (AGly), beta-lactams (Bla), tetracyclines (Tet), phenicols (Phe), macrolides, lincosamides, and streptogramins (MLS), trimethoprim (Tmt), sulfonamides (Sul), fluoroquinolones (Flq), bacitracin (BacA), polymixins (Colistin), fosfomycin (Fcyn), and rifamycin (Rif)]. Beta-lactams were the most diverse drug class with 48 unique ARGs reported followed by the aminoglycosides (*n* = 46), MLS (*n* = 25), tetracyclines (*n* = 23), trimethoprim (*n* = 15), phenicols (*n* = 11), and remaining drug classes had less than 10 unique ARGs for each class. Four samples had no detections of ARGs which were reported at site 1 in the south room on sample collection time point 77, site 5 from both rooms on sample collection time point 1, and site 5 in the west room on sample collection time point 42.

### 3.2 Frequency of ARGs and integron genes by drug class

**FIGURE 1:**
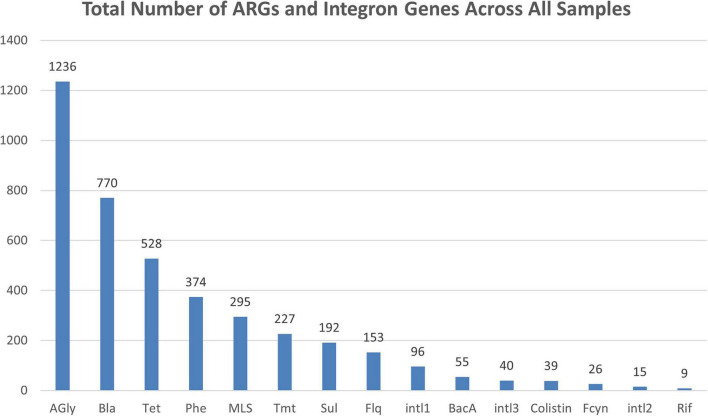
This bar chart represents the total number of ARGs and integron genes detected over all samples from six sites (*n* = 119). There were 184 unique ARGs and 3 classes of integron genes among the 3,904 detections. ARGs and integrons were recorded as a presence or absence of a gene, not relative abundance of the genes within a sample. Total numbers represent accumulation of presence of reported genes from the six sites. AGly, aminoglycosides; Bla, beta-lactams; Tet, tetracyclines; Phe, phenicols; MLS, macrolides, lincosamides, streptogramins; Tmt, trimethoprim; Sul, sulfonamides; Flq, fluoroquinolones; intl1, class 1 integron gene; Bac A, bacitracin; intl3, class 3 integron gene; Colistin, colistin; Fcyn, fosfomycin; intl2, class 2 integron gene; Rif, rifamycin.

When evaluating the total frequency of ARGs out of the 119 water line samples, aminoglycoside ARGs were the most frequently reported (Figure 1). They were followed by beta-lactams, tetracyclines, phenicols, macrolides, lincosamides, and streptogramins, trimethoprim, fluoroquinolones, and other remaining drug classes or integron genes.

### 3.3 Frequency of unique ARGs and integron genes

There were 21 unique ARGs present in at least 50% of the samples (*n* = 119). The remaining 163 unique ARGs were reported in less than 50% of the samples. Table 1 demonstrates the most frequently reported ARGs and integron genes that were present in over 50% of the samples. *SulI* was the most frequently reported gene and was detected in 84% of the samples (Table 1). Integron gene *intl1* was detected in almost 81% of the samples. Although genes associated with colistin resistance were not detected in greater than 50% of the samples, given their significance, it is worth noting the genes reported and their relative frequencies in the samples. Gene *mcr7* was detected in 23 samples (19.33%), *mcr4* was detected in 13 samples (10.92%), and *mcr3* was detected in 3 samples (2.52%). All unique ARG and integron gene names and total number of samples that were detected can be found in the Supplementary Table 1.

**TABLE 1:** Table presents the ARG and integron genes found in greater than or equal to 50% of the samples (*n* = 119).

ARG/integron gene name	Drug/integron class	Number of samples with the ARG or integron gene present out of 119 samples	% of total samples with ARG/integron gene present
*sulI*	Sulfonamide	100	84.03%
*IntI1*	Integron class 1	96	80.67%
*aadA*	Aminoglycoside	95	79.83%
*strB*	Aminoglycoside	94	78.99%
*strA*	Aminoglycoside	94	78.99%
*tetG*	Tetracycline	90	75.63%
*aph3*′′*Ia*	Aminoglycoside	90	75.63%
*floR*	Phenicol	85	71.43%
*aacaad*	Aminoglycoside	85	71.43%
*sulII*	Sulfonamide	84	70.59%
*aac3-Iva*	Aminoglycoside	83	69.75%
*OXA-2*	Beta lactam	77	64.71%
*TEM-1D*	Beta lactam	71	59.66%
*aph4-Ia*	Aminoglycoside	71	59.66%
*aadB*	Aminoglycoside	64	53.78%
*OXA-22*	Beta lactam	62	52.10%
*CARB*	Beta lactam	62	52.10%
*tet*A	Tetracycline	61	51.26%
*aphA2*	Aminoglycoside	61	51.26%
*tetX*	Tetracycline	60	50.42%
*tetC*	Tetracycline	60	50.42%
*cmr*	Phenicol	60	50.42%

### 3.4 Presence of ARGs over collection time points and sites

**FIGURE 2:**
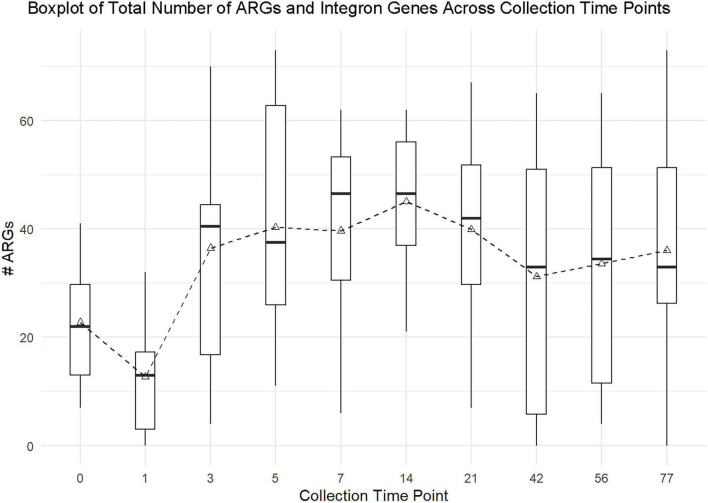
The relationship between 10 collection time points and the total number of unique ARGs and integron genes is shown in the box-and-whisker plot, with dashed lines connecting the means across sample collection time points. Each box represents data collected from 119 samples from the six sites. There was a significant difference in the number of total ARGs and integron genes combined across the collection time points (*p*-value = 0.00). *Post hoc* pairwise comparisons with Tukey’s adjustment demonstrated that ARGs and integron genes were significantly lower on average by 10 unique ARGs/ integron genes 24-h post-treatment (1) when compared to pre-treatment (0) counts (*p*-value = 0.01). Pre-treatment (0) counts on average were not statistically different than most post-treatment sample collection dates (3, 5, 7, 21, 42, 56, and 77) (adjusted *p*-value ≥ 0.05). However, post-treatment collection time point 14 demonstrated a significant difference when compared to pre-treatment (0) with averages being significantly higher on post-treatment collection time point 14.

When evaluating abundance of unique ARGs and integron genes over time, ARGs and integron genes 24-h post-treatment (1) were significantly lower on average by 10 genes from pre-treatment counts (0) (*p* = 0.01) (Figure 2).

ANOVA demonstrated that there was a statistical difference in the number of total unique ARGs among the collection time points (*p*-value = 0.00) (Figure 2). *Post hoc* pairwise comparisons using Tukey’s adjusted method revealed that the total number of unique ARGs and integron genes combined at pre-treatment (0) were not significantly different than most post-treatment collection dates (3, 5, 7, 21, 42, 56, and 77) (adjusted *p*-value ≥ 0.05). However, total number of unique ARGs and integron genes pre-treatment (0) were significantly different from collection time point 14 (adjusted *p*-value = 0.012), with total number of unique ARGs and integron genes on collection time point 14 being higher on average than pre-treatment (0). Total number of unique ARGs and integron genes 24-h post-treatment (1) were significantly lower on average compared to time points 3, 5, 7, 14, 21, 56 and 77.

Changes in relative abundance of unique ARGs encoding for specific drug classes and integron gene classes combined were assessed and no statistical differences over collection time points were found.

## 4 Discussion

This study identified 184 types of unique ARGs associated with resistance to 14 drug classes, including those critically and medically important to both human and livestock health, and three classes of integron genes from swine water line biofilms. Over the entire study, a total of 3,904 ARGs and 151 integron genes were identified. The null hypothesis was rejected that there were no statistical differences in ARGs before and after application of 0.78% PAA. Presence of ARGs and integron genes demonstrated a statistical difference between pre-treatment (0) and 24-h post-treatment (1) after 0.78% PPA application, averaging 10 unique ARG/integron gene reduction compared to pre-treatment. However, the longitudinal effects of 0.78% PAA water line application on presence of ARGs was evaluated and demonstrated rapid reoccurrence of ARGs and integron genes. Post-treatment dates 3, 5 and 7 were not significantly different than pre-treatment (0), with post-treatment time point 14 demonstrating significantly higher presence of unique ARGs and integron genes compared to pre-treatment (0). This demonstrates that one-time administration of 0.78% PAA initially reduced the total number of unique ARGs and integron genes in swine water lines, but long term reductions in ARG and integron gene diversity are not appreciated. The results from this study provide evidence that naturally occurring biofilms in swine water lines harbor resistance gene determinants and provide novel insights into AMR surveillance on swine farms. These findings also demonstrate that PAA can impact prevalence of ARGs, but further research in biofilm management strategies and reduction of ARGs and integron genes long-term is necessary.

To the authors’ knowledge, this is the first documented characterization of the presence of ARGs and integron genes recorded for swine drinking water line biofilms before and after water line cleaning with PAA. While a previous report identified ARGs from a single swine water line biofilm sample reporting ARGs from fewer drug classes (Doughan et al., 2022), the present study demonstrated longitudinal prevalence with greater diversity of ARGs, drug classes, and integron genes from several swine facilities. Several investigations of ARGs on swine farms have primarily focused on their contribution to environmental and foodborne ARGs dissemination, emphasizing the associated risks to human health (Brooks et al., 2014; Gao et al., 2020; He et al., 2019; Li et al., 2016; Scicchitano et al., 2024; Song et al., 2021). In contrast, this study assesses ARGs and integron genes in water line biofilms, which could have implications on pig health, however, the risk remains unknown. This study provides critical insights into the potential sources and pathways of ARG spread through the farm environment, contributing to a broader understanding of ARG transmission in swine farms. A similar study evaluating the presence of ARGs in an organic poultry farm’s water distribution system via next generation sequencing found presence of ARGs associated with resistance to critically important drug classes (Piccirillo et al., 2024). The poultry study found fewer ARGs with a narrower range of drug classes than the present study. The lower prevalence of ARGs and narrower range of drug classes in the poultry study could be attributed to the limited use of antimicrobials in organic farming, or differences between poultry and swine water line biofilm composition and ecology. Alternatively, in the present study, resistances to antimicrobials that are currently not utilized in United States swine production, such as colistin and fosfomycin, were identified. The presence of these ARGs could be attributed to the spread of multi-drug-resistant plasmids containing mobile colistin resistance (*mcr*) genes associated with resistance to colistin. Therefore, absence of use of certain antimicrobials may not entirely eliminate the risk of the presence of ARGs as multiple drug resistance can naturally occur through mutations, spread through the environment and HGT, or spread from bacteria from other countries that utilize colistin in swine production (Hayer et al., 2022). The comparison between studies exposes future research opportunities to further understand ARG prevalence and transmission dynamics and differences in microbiome compositions across multiple types of production systems.

Antimicrobial resistance gene or integron gene presence in this study was not linked to specific bacterial species, microbiome compositions assessed, genetic context of ARGs on transposons or plasmids, nor relative abundance of specific genes estimated within samples. Future studies could employ quantitative polymerase chain reaction (qPCR) to identify and quantify specific ARGs in bacterial cultures, or leverage high-throughput genomic and epigenomic techniques, such as Hi-C, to identify specific bacterial carriers of clinically significant ARGs in water line biofilm samples (Li et al., 2021). For example, one Iranian study evaluated tetracycline resistance genes from *Escherichia coli* (*E. coli*) isolates from water line biofilms in poultry farms (Ahangaran et al., 2022). This study found multiple efflux pump-related genes encoding resistance to tetracyclines, such as *tetA* and *tetB* genes, from *E. coli* cultures and subsequent PCR (Ahangaran et al., 2022). Further metagenomic assessment in swine water line biofilm composition and identification of mobile genetic elements could provide relevant microbiological ecological insights and should be utilized in future studies. Co-location of ARGs on mobile genetic elements has been described for several ARGs detected in this study (Bhat et al., 2023; Domingues et al., 2015; Gillings, 2014; Partridge et al., 2009, 2018), although genomic context and co-location were not evaluated and therefore, their arrangements cannot be confirmed. Additionally, conducting quantitative microbial risk assessments utilizing these technologies would provide valuable insights into the potential health risks posed to both humans and animals (Li et al., 2021).

However, even without knowledge of ARGs associated with specific bacterial species, several medically important ARGs were detected in water line biofilms in this study (Scott et al., 2019; World Health Organization [WHO], 2024). The first high-priority group of ARGs included those associated with resistance to colistin (polymyxin E), which is considered a last resort antimicrobial against multiple-drug-resistant gram-negative bacteria in humans. Notably, colistin is not available for use in swine veterinary medicine in the United States (Guo et al., 2024). Three colistin ARGs were detected (i.e., *mcr-3*, *mcr-4*, *mcr-7*) also known as *mcr* enzymes, which are plasmid-mediated, facilitating potential horizontal gene transfer (HGT) (Guo et al., 2024). Beta-lactam resistance had the most unique ARGs (i.e., 48) with 34 of these genes being OXA beta-lactamase ARGs. OXA beta-lactamases were some of the first beta-lactamases identified and have been named in the chronological order they were identified. They were initially limited to penicillins, but throughout time, specific OXA genes have expanded to cephalosporins, extended-spectrum beta-lactamases (ESBL), and carbapenems (i.e., *OXA-48*) (Evans and Amyes, 2014). An in-depth analysis of OXA beta lactamases has been reviewed elsewhere (Evans and Amyes, 2014). Carbapenems are also a medically important, last resort antimicrobial with human health implications (Papp-Wallace et al., 2011). Additional ARGs were also detected which have associated resistance to carbapenems and ESBL, such as *AIM-1*, *bla*_BKC_, *CARB-5*, *CARB*, *JOHN-1*, and more. Six ARGs were detected to have associated resistance to fluoroquinolones, *qnrB*, *qnr*-*S*, *qnrVC1*, *oqxA*, *oqxB*, *qepA*, where *qnr* genes are commonly found in *Enterobacteriaceae* sp. and can be found in the same plasmid conferring resistance to ESBL (Salah et al., 2019). ARGs *oqxA*, *oqxB*, and *qepA* are efflux pumps which also work primarily against fluoroquinolones (Amereh et al., 2023; Yamane et al., 2008). Other ARGs detected in this study that encode resistance to medically important antimicrobials include aminoglycosides, the most frequently detected ARGs found in the present study, (i.e., *strA*, *strB*, *aph3*′′*Ia*, *aadA*, *aacaad*, etc.), ansamycins (rifamycin – *arr4*), phosphonics such as fosfomycin (i.e., *fosA*, *fos2A*), phenicols (i.e., *catB2*, *catBx*, *cmlA*, *cmr*, *floR*, etc.), macrolides, lincosamides, and streptogramines (i.e., *ermF*, *ermB*, *mphE*, *oleC*, *mrsE*, etc.), tetracyclines (i.e., *tetG*, *tetX*, *tetC*, etc.), sulfonamides (i.e., *sulI*, *sulII*, *sulIII*), and trimethoprim (i.e., *dfrA*, *dfrA1*, *dfrA5*, etc.). The presence of ARGs and integron genes in swine water lines with associated resistance to medically important antimicrobials pose a risk of further horizontal gene transfer to pathogens or zoonotic bacteria.

Although the one-time application of 0.78% of PAA had a significant impact on unique ARGs and integron genes immediately post-treatment (1), long-term reductions in unique ARGs were not appreciated. Total number of ARGs and integron genes combined from pre-treatment (0) were not statistically different than most post-treatment collection time points (3, 5, 7, 21, 42, 56, and 77). Collection time point 14 was significantly different than pre-treatment totals (*p*-value = 0.0127) with pre-treatment (0) values estimated to be 22.33 ARGs and integron genes lower on average than sample collection time point 14 values. This demonstrates a visual and statistical trend that unique ARGs did increase on average after post-treatment (1) and achieved significantly higher unique ARGs and integron gene numbers by collection time point 14 (Figure 2). This trend requires further mechanistic investigation. The dose of 0.78% PAA, compared to the 2% labeled dose for CID 2000 Pro, could have resulted in an ineffective elimination of bacteria, increasing selective pressure for factors involved in bacterial survival, and inadvertently selecting for organisms that carry ARGs or integron genes (Curiao et al., 2016; Maillard and Centeleghe, 2023; Webber et al., 2015). Furthermore, the use of biocides can aid in the co-selection of ARGs and resistance mechanisms against biocides (Chapman, 2003; Webber et al., 2015). This provides relevant guidance that water line cleaning and disinfection with PAA at 0.78% may significantly reduce ARGs and integron genes in the short term but may have greater efficiencies with the labeled dose for complete organism inactivation. Studies have assessed the effect of PAA on functionality of ARGs and have found that although it is effective on microorganisms, it does not have great efficacy at reducing the functionality or removal of ARGs left behind (Yin et al., 2023; Zhang et al., 2019). This suggests that other complementary disinfectant mechanisms may be necessary for complete ARG removal, such as ultraviolet light irradiation (Zhang et al., 2019).

Detecting ARGs through sequencing can be a useful tool for their surveillance and provides insights into their epidemiology (Maddock et al., 2024). However, the presence of ARGs and integron genes does not directly correlate with phenotypic expression, the viability of the functionality of the genes, or direct clinical relevance (Maddock et al., 2024). Phenotypic demonstration of resistance is essential to interpret this in the sense of aiding the selection of treatment or predicting how a bacteria may respond to treatment (Maddock et al., 2024). In order to demonstrate true clinical implications, resistance genes must also be associated with a clinically relevant pathogen. Resistance genes can be associated with non-pathogenic bacteria, thus not requiring treatment (Deekshit and Srikumar, 2022; Maddock et al., 2024). The risk of the presence of ARGs found in this present study is that ARGs and integron genes could be spread to potential pathogens either located in the water line biofilms, in the pig, or in the pig’s environment via the drinking water source. The spread of these ARGs to clinically relevant pathogens through HGT or other mechanisms could equip them to potentially demonstrate phenotypic resistance and contribute further to AMR (Deekshit and Srikumar, 2022). Future research in the detection and evaluation of phenotypic resistance expression in swine water line biofilms is necessary.

Due to the nature of the longitudinal study and to ensure proper animal health and welfare, water administered antimicrobials, electrolytes, and oral live attenuated vaccines were permitted to be administered in the water lines throughout the study under veterinary guidance. No water line cleaners or disinfectants were permitted to be run in the water lines after the 0.78% PAA treatment. It was outside the scope of the study to determine the impacts of subsequent water line administered products and their effect on presence of ARGs and integron genes, especially after antimicrobial use. Additionally, identification and classification of ARGs was subject to the comparison to the ARGannot_r3 database, and the identification coverages set within the pipeline for proper identification. Genes were assigned based on the database; however, some genes have been recognized to also work against other drug classes. This is a limitation of the study as a broad review to connect resistance genes with all possible drug classes they may be effective against was not conducted. Furthermore, continual evolution and identification of new ARGs is a moving target. It is possible that at the time of this analysis, ARGs that have not been recorded are still present, leading to underrepresentation of ARGs in the results.

## Data Availability

The datasets generated for this study can be found in the National Library of Medicine National Center for Biotechnology Information (NCBI) BioProject database at this link: https://www.ncbi.nlm.nih.gov/bioproject/1331925.
