# Supplementary material for: Presence of antimicrobial resistance genes in biofilms from swine drinking water pipes before and after treatment with peracetic acid
Source: Front Microbiol. 2026 Apr 29;17:1770950. doi: 10.3389/fmicb.2026.1770950 (PMC13168167; doi:10.3389/fmicb.2026.1770950)
Supplement: Supplementary file 1 [file Table_1.docx]

***Supplementary material***

1. **Supplementary Figures and Tables**

S1. Percent Presence of ARGs/ Integron Genes in All Samples (n=119)

| **ARG / Integron gene name** | **Drug Class / Integron Class** | **Number samples ARG present** | **% present in total samples** |
| --- | --- | --- | --- |
| *sulI* | Sul | 100 | 84.03% |
| *intI1* | Integron class 1 | 96 | 80.67% |
| *aadA* | AGly | 95 | 79.83% |
| *strB* | AGly | 94 | 78.99% |
| *strA* | AGly | 94 | 78.99% |
| *tetG* | Tet | 90 | 75.63% |
| *aph3''Ia* | AGly | 90 | 75.63% |
| *floR* | Phe | 85 | 71.43% |
| *aacaad* | AGly | 85 | 71.43% |
| *sulII* | Sul | 84 | 70.59% |
| *aac3-Iva* | AGly | 83 | 69.75% |
| *OXA-2* | Bla | 77 | 64.71% |
| *TEM-1D* | Bla | 71 | 59.66% |
| *aph4-Ia* | AGly | 71 | 59.66% |
| *aadB* | AGly | 64 | 53.78% |
| *OXA-22* | Bla | 62 | 52.10% |
| *CARB* | Bla | 62 | 52.10% |
| *tetA* | Tet | 61 | 51.26% |
| *aphA2* | AGly | 61 | 51.26% |
| *tetX* | Tet | 60 | 50.42% |
| *tetC* | Tet | 60 | 50.42% |
| *cmr* | Phe | 60 | 50.42% |
| *aph* | AGly | 56 | 47.06% |
| *oqxB* | Flq | 55 | 46.22% |
| *catB2* | Phe | 55 | 46.22% |
| *bacA* | Bacitracin | 55 | 46.22% |
| *catBx* | Phe | 54 | 45.38% |
| *OXA-60* | Bla | 52 | 43.70% |
| *ermF* | MLS | 51 | 42.86% |
| *aac6-32* | AGly | 51 | 42.86% |
| *dfrA1* | Tmt | 49 | 41.18% |
| *aadA4-5* | AGly | 49 | 41.18% |
| *msrE* | MLS | 46 | 38.66% |
| *dfrA* | Tmt | 45 | 37.82% |
| *tet-42* | Tet | 43 | 36.13% |
| *mphE* | MLS | 42 | 35.29% |
| *qepA* | Flq | 41 | 34.45% |
| *oleC* | MLS | 41 | 34.45% |
| *aac6-Iz* | AGly | 41 | 34.45% |
| *tet-36* | Tet | 40 | 33.61% |
| *intI3* | Integron class 3 | 40 | 33.61% |
| *dfrA5* | Tmt | 37 | 31.09% |
| *AIM-1* | Bla | 36 | 30.25% |
| *cmlA* | Phe | 33 | 27.73% |
| *oqxA* | Flq | 31 | 26.05% |
| *aac3-Ii* | AGly | 28 | 23.53% |
| *CAU* | Bla | 27 | 22.69% |
| *aac3-IIa* | AGly | 27 | 22.69% |
| *tet-34* | Tet | 26 | 21.85% |
| *OXA-12* | Bla | 25 | 21.01% |
| *aph2''Ic* | AGly | 25 | 21.01% |
| *SHV-OKP-LEN* | Bla | 24 | 20.17% |
| *dfrB2* | Tmt | 24 | 20.17% |
| *mcr7* | Colistin | 23 | 19.33% |
| *THIN* | Bla | 22 | 18.49% |
| *tet-39* | Tet | 22 | 18.49% |
| *dfrA16* | Tmt | 22 | 18.49% |
| *qnrB* | Flq | 21 | 17.65% |
| *OXA-48* | Bla | 21 | 17.65% |
| *fosA2* | Fcyn | 21 | 17.65% |
| *ermB* | MLS | 21 | 17.65% |
| *CMY* | Bla | 21 | 17.65% |
| *CEPH-AQU* | Bla | 21 | 17.65% |
| *catA2* | Phe | 21 | 17.65% |
| *aac6-Ic* | AGly | 21 | 17.65% |
| *tetE* | Tet | 20 | 16.81% |
| *OXA-1* | Bla | 20 | 16.81% |
| *mrdA* | Bla | 20 | 16.81% |
| *ampH* | Bla | 20 | 16.81% |
| *ACT-MIR* | Bla | 20 | 16.81% |
| *vatF* | MLS | 19 | 15.97% |
| *tetY* | Tet | 18 | 15.13% |
| *tetQ* | Tet | 18 | 15.13% |
| *CEPH* | Bla | 18 | 15.13% |
| *CatB1* | Phe | 18 | 15.13% |
| *aadD* | AGly | 18 | 15.13% |
| *tetB* | Tet | 17 | 14.29% |
| *sat-2A* | AGly | 17 | 14.29% |
| *aac6-IIc* | AGly | 17 | 14.29% |
| *aac3-VIa* | AGly | 17 | 14.29% |
| *aph3-Ib* | AGly | 16 | 13.45% |
| *aac3-Vb* | AGly | 16 | 13.45% |
| *OXA-50* | Bla | 15 | 12.61% |
| *intI2* | Integron class 2 | 15 | 12.61% |
| *tetD* | Tet | 14 | 11.76% |
| *catB7* | Phe | 14 | 11.76% |
| *mphA* | MLS | 13 | 10.92% |
| *mcr4* | Colistin | 13 | 10.92% |
| *cmlB1* | Phe | 13 | 10.92% |
| *aph3-Id* | AGly | 13 | 10.92% |
| *OXA-211* | Bla | 12 | 10.08% |
| *ereA* | MLS | 12 | 10.08% |
| *dfrA19* | Tmt | 12 | 10.08% |
| *AMPH* | Ecoli | 12 | 10.08% |
| *OXA-209* | Bla | 11 | 9.24% |
| *catA1* | Phe | 11 | 9.24% |
| *tetL* | Tet | 10 | 8.40% |
| *mefA* | MLS | 10 | 8.40% |
| *dfrB* | Tmt | 10 | 8.40% |
| *catB10* | Phe | 10 | 8.40% |
| *ampC1* | Ecoli | 10 | 8.40% |
| *OXA-237* | Bla | 9 | 7.56% |
| *L-1* | Bla | 9 | 7.56% |
| *cfxA* | Bla | 9 | 7.56% |
| *arr4* | Rif | 9 | 7.56% |
| *aac3-Ib* | AGly | 9 | 7.56% |
| *tetM* | Tet | 8 | 6.72% |
| *sulIII* | Sul | 8 | 6.72% |
| *ORN-PLA* | Bla | 8 | 6.72% |
| *dfrA8* | Tmt | 8 | 6.72% |
| *dfr* | Tmt | 8 | 6.72% |
| *aph3-IIb* | AGly | 8 | 6.72% |
| *aac3-Ic* | AGly | 7 | 5.88% |
| *spc* | AGly | 6 | 5.04% |
| *LCR* | Bla | 6 | 5.04% |
| *aph3-III* | AGly | 6 | 5.04% |
| *aph3-IIc* | AGly | 6 | 5.04% |
| *aac6-33* | AGly | 6 | 5.04% |
| *aac3-Ih* | AGly | 6 | 5.04% |
| *tetW* | Tet | 5 | 4.20% |
| *PER-1* | Bla | 5 | 4.20% |
| *OXA-228* | Bla | 5 | 4.20% |
| *mphB* | MLS | 5 | 4.20% |
| *fosA* | Fcyn | 5 | 4.20% |
| *aac3-I* | AGly | 5 | 4.20% |
| *tetZ* | Tet | 4 | 3.36% |
| *sat4A* | AGly | 4 | 3.36% |
| *qnrVC1* | Flq | 4 | 3.36% |
| *PER-6* | Bla | 4 | 3.36% |
| *OXA-7* | Bla | 4 | 3.36% |
| *OXA-42* | Bla | 4 | 3.36% |
| *OXA-18* | Bla | 4 | 3.36% |
| *OXA-114* | Bla | 4 | 3.36% |
| *lnuA* | MLS | 4 | 3.36% |
| *linB* | MLS | 4 | 3.36% |
| *ermX* | MLS | 4 | 3.36% |
| *aadA14* | AGly | 4 | 3.36% |
| *aac* | AGly | 4 | 3.36% |
| *vatB* | MLS | 3 | 2.52% |
| *tet-33* | Tet | 3 | 2.52% |
| *tet-31* | Tet | 3 | 2.52% |
| *pam* | Bla | 3 | 2.52% |
| *msrD* | MLS | 3 | 2.52% |
| *mcr3* | Colistin | 3 | 2.52% |
| *mbl* | Bla | 3 | 2.52% |
| *lnuD* | MLS | 3 | 2.52% |
| *ermC* | MLS | 3 | 2.52% |
| *dfrC* | Tmt | 3 | 2.52% |
| *dfrA10* | Tmt | 3 | 2.52% |
| *aadA9* | AGly | 3 | 2.52% |
| *aac3-Id* | AGly | 3 | 2.52% |
| *tet-40* | Tet | 2 | 1.68% |
| *tet-37* | Tet | 2 | 1.68% |
| *OXA-58* | Bla | 2 | 1.68% |
| *lnuF* | MLS | 2 | 1.68% |
| *lnuC* | MLS | 2 | 1.68% |
| *erm36* | MLS | 2 | 1.68% |
| *dfrB4* | Tmt | 2 | 1.68% |
| *dfrA27* | Tmt | 2 | 1.68% |
| *CARB-5* | Bla | 2 | 1.68% |
| *ant6-Ia* | AGly | 2 | 1.68% |
| *ampC* | Bla | 2 | 1.68% |
| *aac3-III* | AGly | 2 | 1.68% |
| *tetO* | Tet | 1 | 0.84% |
| *tetJ* | Tet | 1 | 0.84% |
| *SRT-SST* | Bla | 1 | 0.84% |
| *qnr-S* | Flq | 1 | 0.84% |
| *POM* | Bla | 1 | 0.84% |
| *OXY* | Bla | 1 | 0.84% |
| *OXA-214* | Bla | 1 | 0.84% |
| *mefB* | MLS | 1 | 0.84% |
| *lsaB* | MLS | 1 | 0.84% |
| *JOHN-1* | Bla | 1 | 0.84% |
| *FAR-1* | Bla | 1 | 0.84% |
| *ermG* | MLS | 1 | 0.84% |
| *ermA* | MLS | 1 | 0.84% |
| *erm42* | MLS | 1 | 0.84% |
| *dfrA3* | Tmt | 1 | 0.84% |
| *dfrA20* | Tmt | 1 | 0.84% |
| *BKC* | Bla | 1 | 0.84% |
| *BES-1* | Bla | 1 | 0.84% |
| *APH-Stph* | AGly | 1 | 0.84% |
| *aph2-Ib* | AGly | 1 | 0.84% |
| *ant6-Ib* | AGly | 1 | 0.84% |
| *aadC* | AGly | 1 | 0.84% |
| *aac6-Im* | AGly | 1 | 0.84% |
| *aac6-Id* | AGly | 1 | 0.84% |

**Key:** AGly, aminoglycosides; Bla, beta-lactams; Tet, tetracyclines; Phe, phenicols; MLS, macrolides, lincomycins, streptogrammins; Tmt, trimethoprim; Sul, sulfonamides; Flq, fluoroquinolones; Fcyn, Fosfomycin; Rif, rifamycin.
